# Contrastive Lexical Diffusion Coefficient: Quantifying the Stickiness of the Ordinary

**DOI:** 10.1145/3442381.3449819

**Published:** 2021-04

**Authors:** Mohammadzaman Zamani, H. Andrew Schwartz

**Affiliations:** Stony Brook University, New York, USA

**Keywords:** Lexical Diffusion, Ordinary Language, Diffusion Model, Language Change

## Abstract

Lexical phenomena, such as clusters of words, disseminate through social networks at different rates but most models of diffusion focus on the discrete adoption of new lexical phenomena (i.e. new topics or memes). It is possible much of lexical diffusion happens via the changing rates of existing word categories or concepts (those that are already being used, at least to some extent, regularly) rather than new ones. In this study we introduce a new metric, *contrastive lexical diffusion* (*CLD*) *coefficient*, which attempts to measure the degree to which ordinary language (here clusters of common words) catch on over friendship connections over time. For instance topics related to meeting and job are found to be sticky, while negative thinking and emotion, and global events, like ‘school orientation’ were found to be less sticky even though they change rates over time. We evaluate CLD coefficient over both quantitative and qualitative tests, studied over 6 years of language on Twitter. We find CLD predicts the spread of tweets and friendship connections, scores converge with human judgments of lexical diffusion (r=0.92), and CLD coefficients replicate across disjoint networks (r=0.85). Comparing CLD scores can help understand lexical diffusion: positive emotion words appear more diffusive than negative emotions, first-person plurals (we) score higher than other pronouns, and numbers and time appear non-contagious.

## 1 INTRODUCTION

Language is and always has been subject to change. For instance, terms can be introduced, vanish, or change in meanings and applications over time [30]. All such changes happen under complex social influences [28], carried out within social networks of people.

**Figure 1:**
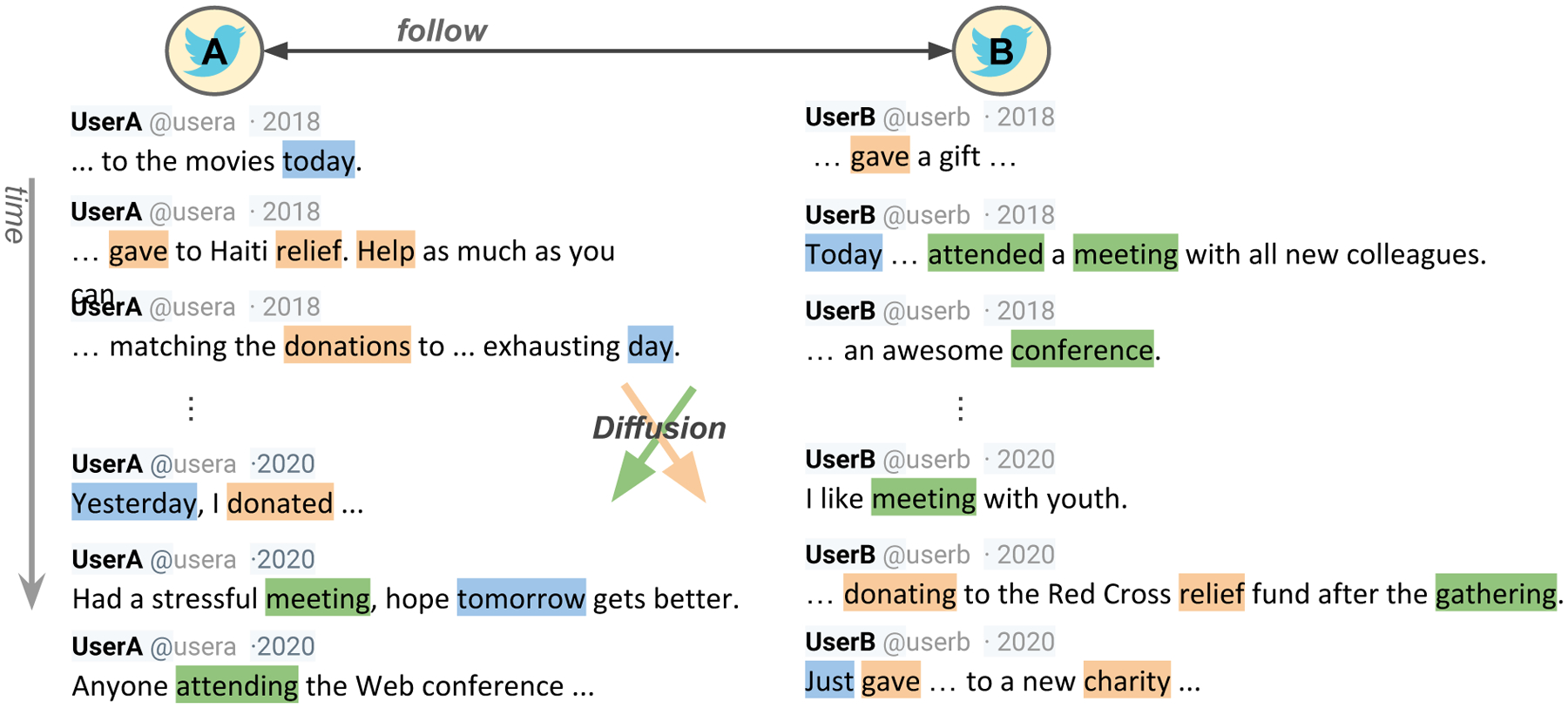
Language can diffuse over common words or topics. In this example, talking about charity (light/orange terms) or attending meetings (dark/green terms) already exist in both user vocabularies but still propagate in frequency across social network connections, while talking about current happenings (blue terms) are common but do not seem to spread.

Researchers are increasingly developing techniques to understand language change in the context of social networks, often termed ‘language diffusion’. Building on models of disease contagion, which are often seen as dichotomous events (infection or not), much of such work focuses on the diffusion of new terms (i.e. the term is either present or not for an individual). For example, researchers have analyzed lexical emergence in Twitter [15, 16], investigated the history of language borrowing between cultures [20], and characterized relationship of lexical influence across demographics and geography [10]. In such studies, language change is operationalized as a process of introducing and spreading new lexical innovations. Few have studied the spread of ordinary language (i.e. that which most people of a particular language use on a regular basis; example given in Figure 1), which leaves a gap in our understanding of diffusion.

This gap is important because a majority of language utterances are in fact of the ordinary. lexical phenomena tend to follow a Zipfian distribution of frequencies where a large portion of the terms we use represent only a small portion of all possible terms [34]. While the long tail itself has been an interesting challenge for NLP, one should not ignore that most word instances come from the head of the distribution.

The change in focus from the tail to the head of the distribution translates into important methodological distinctions: models intended for tracking new phenomena are often ill-suited for tracking diffusion of ordinary terms since they rely heavily on binary or discrete-variable metrics that often do not capture change in frequency by single users or nodes. In analogy to disease infection [13], term diffusion is mostly modeled as a binary outcome. However, measuring more coarse or ordinary lexical phenomena requires tracking change in frequency, and could greatly increase our understanding of language diffusion.

In this study, we propose the *Contrastive Lexical Diffusion (CLD) Coefficient* for quantifying content diffusion of *ordinary* lexical phenomena – lexical categories that make up most of our language and yet there is a gap in our understanding of their diffusion – over time with respect to the effect of friendship networks and accounting for population language. We evaluate this method by applying it to common clusters of words in social media, calculating the CLD coefficient of ordinary LDA topics. Evaluation is facilitated through showing the CLD coefficient (a single feature) is by itself quite predictive for many tasks such as like and retweet prediction as well as guess whether pairs of nodes are friends, in addition to agreement with human judges. Finally, we extend the model to other categories of language, the lexical Inquiry and Word Count categories and examining which common categories are more likely to spread among friends.

## 2 RELATED WORK

The study of language change by itself has a rich history, which most of it has been done in a historical context by analyzing patterns of language change [26, 29, 42]. However, with the availability of network and geographical data in conjunction with language data, a novel view of language change has emerged as a diffusion process of linguistic elements [22]. Our work follows this tradition but produces measurements of the lexical phenomena themselves (i.e. rather than the nodes or network structures) and caters toward common phenomena that make up a large portion of every utterances.

Most previous work on lexical diffusion can be characterized as either studying: (1) new terms or (2) change in meanings of existing words, as well as by its network type: (1) geographical or (2) social/friendship. Importantly, little attention has been given to measuring and understanding the diffusion of ordinary lexical phenomena, which has an advantage of utility in downstream modeling applications since it covers a large area under the Zipfian distribution.

Linguistic variance across geographic units is perhaps the most well documented. For example, Grieve et al. mapped lexical innovations to track the spread of new words on American Twitter [16]. Kulkarni et al. presented an embedding-based technique to detect changes in word meaning across geographical regions [27]. Eisenstein et al. characterized the relationship of linguistic influence to a set of demographic and geographic predictors, suggesting demographic similarity and geographical proximity drives lexical influence [10]. Further, Coviello et al. suggested lexical sentiment is itself contagious across geography [5].

Language itself is a form of information and information often propagates through social ties. For instance, Bakshy et al. studied the role of strong and weak ties in exposure to information diffusion [4]. However, while there are many studies on information propagation on social networks, few have studied language diffusion over friendship networks. De et al. and Ke et al. studied different characteristics of social network and their role in language change [9, 22].

Such works start with a process of capturing innovations – new terms emerging among a group of well-connected individuals. The next step examines diffusion: first, speakers (or writers) must come into contact with the new lexical phenomena; second, they must decide to use it [6, 11]. This diffusion step is studied most extensively, with a wide range of applications: from investigating the history of language borrowing [20] to analyzing forum member life cycles [7].

Most measure diffusion as a function of the number of individuals (or nodes) who mention a specific phenomenon (rather than frequency of the phenomena), so it is perhaps intuitive that there are few works examining phenomena frequency. However, there are some exceptions. For example, frequency has been used to estimate latent trajectories of phenomena [31] and to capture differing semantics across geographical regions [27], but both of these are fundamentally different tasks than contagion of a lexical phenomenon (we use trajectories as a baseline). In this paper, we took a rather unique path compared to the majority of the body of research: (1) we investigate language diffusion with focus on usage *frequency* of ordinary language units over people, and (2) we characterize *language phenomena* (here, clusters of common words) using social networks rather than characterizing networks/nodes using language.

## 3 DATASET

Language is a social behavior and each word we write or speak can be seen as a social activity [25], and different behaviors and activities can have various effect on our social ties and friends [39]. Taking behavior to be mention of a common topic, we utilize a dataset to reflect these points. We derived two social networks from a population of 174k Twitter users, by starting from two sets of random twitter users, of size 2 and 1, and collecting users with distance of at most 3 to any of them. Those within 2 hops distance of our starting users (all for whom anyone who is both their follower and followee is among the population) were considered *core* users of the sample. We acquired two independent networks of 3, 298 *core* users (development data) and 978 *core* users (replication data). Such data was directly scraped from Twitter using Twitter API.

For each population user, we extracted at most 500 (non-retweet) tweets, including the text, creation date, number of likes and number of retweets, all written in English. This gave us a pool of 83 million original (non-retweet) tweets, split into two time spans with roughly equal number of tweets. 40 million created between 2013 to 2016 – our data for *past* time span (see method), and 43 millions created between 2017 to 2018 – our data for *current* time span.

## 4 METHOD

Frequency of language units change over time within a social network. We distinguish the individual, friends of the individual, and the general network population, in order to measure diffusion of language units themselves. We view this measure as defining a specific property of language units (rather than network nodes) that characterizes how much it “sticks” (or propagates) specifically over direct connections.

Broadly, our approach is two fold. First, we measure language usage of people and their friends at two different time spans. Then, we compare language usage over the time spans, and in comparison to friends versus the general population. To capture language usage, we consider lexical features of the individual documents generated by the individual within the two time spans.

While our method may generalize to many features, we focus on lexical categories, specifically clusters of semantically related words (topics). As the clusters themselves are not a focus of this work, we downloaded a pre-existing set of 2000 social media clusters extracted using Latent Dirichlet Allocation [37]. Such topics have previously been applied to other social media documents including Twitter settings [35, 41]. Applying the topics to our data leaves us with 2, 000 feature frequencies per user. While the topics themselves are already somewhat ordinary lexical units, we further restrict analysis to the 500 most common. With topics as features, we build language usage matrix *L*_*n×m*_ where *n* is the number of individuals, *m* is the number of topic features, and *L*_*ij*_ is the value of topic *j* for individual *i*.

### Measuring Language Change.

We split each individuals’ documents (e.g. tweets) into 2 time spans, *past*: 2013–2016 and *current*: 2017–2018. For each individual, we then aggregate the features into the time spans, building *L*^*p*^ and *L*^*c*^ as language usage matrices for *past* and *current* respectively. Language change is then defined as Δ*L* = *L*^*c*^ − *L*^*p*^.^1^

Having a vector representation of language change for the individual, i.e. Δ*L*, we then consider his/her friends’ language. Specifically, we calculate a vector for individual i*’s* friends, *F*_*i*_, by averaging over friends’ language usage:

(1) $$F_{i}=\frac{\Sigma_{j\in fr\left(i\right)}L_{j}}{\left|fr\left(i\right)\right|}$$

where, *L*_*j*_ is the *j*^*th*^ row of language usage matrix *L*, *fr*(*i*) is the set of individual *i*’s friends, and |*fr*(*i*)| is the cardinality of the set *fr*(*i*). Now, we can define friends’ language usage matrix *F*_*n×m*_ by vertically concatenating {*F*_*i*_}:

(2) $$F=\left[F_{1};F_{2};\cdots ;F_{n}\right]$$

To arrive at a measure of lexical diffusion, as we will explain next, we need to compare the individual’s change in language to the friends’ past language (i.e. the friend’s language is *before* the change). This mirrors causal modeling in that the cause, here friends language, happens prior to its effect, here users’ *current* language [3]. Thus, matrix *F* is only derived for the *past* time span, and Δ*F* = *F*−*L*^*p*^ is thus the friends’ language difference.

### Contrastive Lexical Diffusin (CLD) Coefficient.

After measuring language change we have two differences: (1) that between the individual in the *past* and *current* (Δ*L*) and (2) that between the individual in the *past* and their friends in the *past* (Δ*F*). Thus, if a particular lexical unit was catchy you would expect its Δ*L* and Δ*F* to covary, on average.

To find how much individual’s language evolve towards their friends’ language, we measure the similarity of Δ*F* with Δ*L* *per feature*. We look at the *Delta* matrices column-wise and obtain the similarity of columns of these two matrices. For instance, Δ*L*^j^, which is *j*^*th*^ column of matrix Δ*L*, represents change in topic *j* from *past* to *current*. Similarly, the Δ*F*^*j*^ is *j*^*th*^ column of Δ*F* represents friends’ language difference for topic *j*. Therefore, for each topic the similarity between vectors Δ*F*^j^ and Δ*L*^j^ shows the extent the usage of topic *j* has changed from *past* to *current* towards friends’ language. So, we need to exploit a measure to capture similarity between vectors. We use cosine similarity since the dimensions of these vectors represent individuals and we need a similarity measure that cannot be overtaken by just a few dimensions. Additionally, our vectors do not simply represent a point, but rather vectors that share an origin *i.e. L*^*p*^.

While the similarity between Δ*F* and Δ*L* represents how much the individual’s language is changing toward their friends, it is possible that the individual is just matching a population-wide trend which is not specifically diffused through their friends. Thus, we control for language use of the whole population, the average language usage of all of the individuals, can be subject to change in the same way we defined friends’ language effect. We define vector *p* as follows:

(3) $$p=\frac{\Sigma_{j}L_{j}}{n}$$

where, *L*_*j*_ is the *j*^*th*^ row of language usage matrix L, and *n* is the number of individuals in the population. The population’s language usage matrix *P*_*n*×*m*_ is obtained by horizontally concatenating *p*, n times. The population’s language is the same no matter which individual we are calculating.

We calculate population’s effect by finding how population’s *current* language differs from individual’s *past* language, as Δ*P* = *P* − *L*^*p*^. Unlike friends’ effect we want to consider population’s language at *current* time span to measure how much of individual’s language change deviated from population toward friends’ effect. Finally, for each topic *j* the cosine similarity between vectors Δ*P*^*j*^ and Δ*L*^*j*^ shows how much the change in usage of topic *j* over time is towards population’s language.

Finally, for each topic *j* we quantify how much Δ*L*^*j*^ changes more towards friends than towards population by comparing the two its cosine similarities. If friend’s similarity score is larger than that of population it means that topic changed more towards friends’ language rather than population’s language. Our final CLD coefficient, *ψ*_*j*_, is thus:

(4) $$\psi_{j}=C\left(\Delta F^{j},\Delta L^{j}\right)-C\left(\Delta P^{j},\Delta L^{j}\right)$$

where *C*(*x*, *y*) is the cosine similarity of vectors *x* and *y*. The higher *ψ*_*j*_ value a topic gets, the more probable to spread it is among friendship relations.^2^

**Figure 2:**
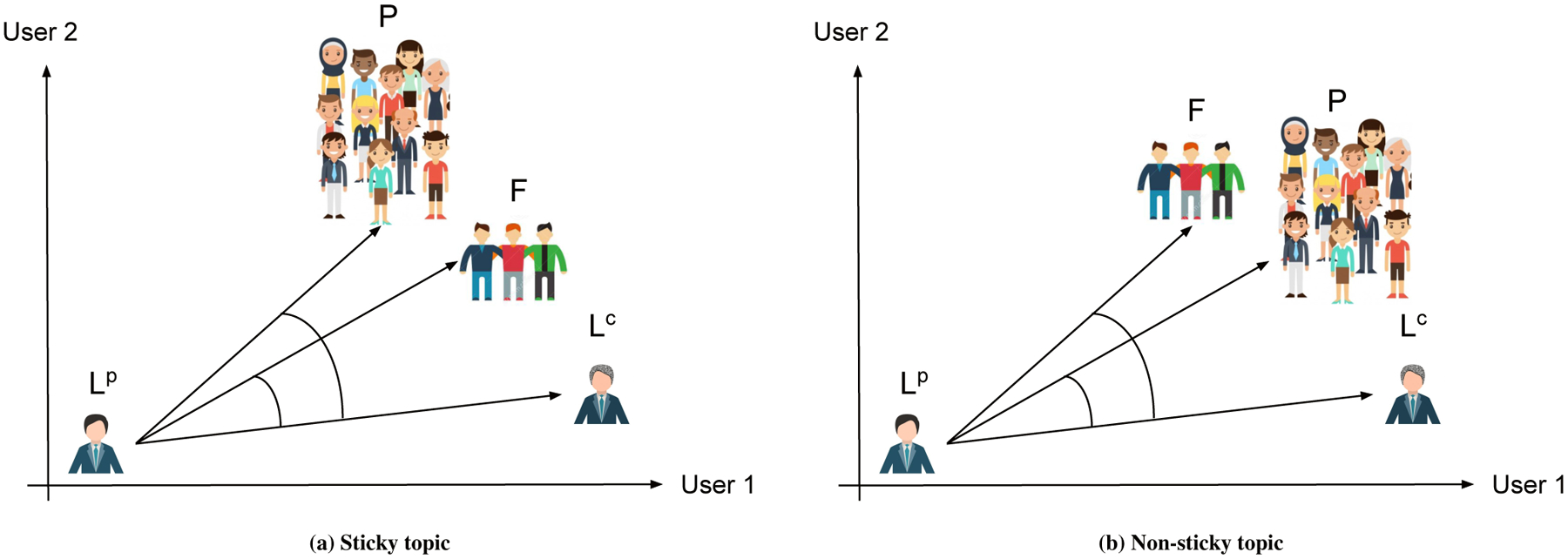
Synthetic example of calculating CLD coefficient of a topic, in a network containing two *core* users. *L*^*p*^ and *L*^*c*^ represent language of *core* users at *past* and *current* time spans. *P* represents language of population and *F* represents language of friends of *core* users.

Figure 2 demonstrates how diffusion score of a topic will be calculated, by showing it for only two *core* users. In this diagram, each axis represents a core user, and since we are plotting the diagram for a topic *t*, the projection of a point on an axis is the normalized usage frequency of topic *t* by the specified user. For instance, *L*^*p*^ is a point in this two dimensional space, where the first dimension is the usage frequency of *t* by user 1 in the *past*, and the second dimension is the usage frequency of *t* by user 2 in the *past*. Similarly, *L*^*c*^ represents topic usage frequency of user 1 and user 2 in *current*. The other two points in this diagram, *F*, and *P*, belong to topic *t* usage frequency for friends at *past* and population at *current* correspondingly. Δ*L* = *L*^*c*^ − *L*^*p*^ represents language change, here, only for topic *t*. $\vec{F}=F-L^{p}$ is friends’ effect and $\vec{P}=P-L^{p}$ is population’s effect. Capturing the angle between $\vec{F}$ and $\vec{L}$, and comparing it with angle between $\vec{P}$ and $\vec{L}$, using cosine similarity, quantifies the extent to which language change is towards friends’ or not, controlling for population. Higher cosine similarity for friends’ effect in compare to population’s suggests the topic is more sticky through friendship communications.

### Relation to causation and influence.

Correlation does not imply causation. In an ideal case, causal inference^3^ utilizes randomized experiments such as a Randomized Controlled Trial(RCT) [21], in which people will randomly be divided into two separate groups: a treatment group and a control group. When the treatment group has no difference from the control except the treatment, the effect of the treatment on the outcome can be assumed to be the difference between the outcome of these two groups.

Unfortunately, randomly assigning “treatments” to people is not feasible nor ethical, rendering RCTs not possible in observational situations like ours as we cannot dictate who is friends with whom in life. However, observational studies need not be limited to simple correlational analyses [24]. Analytic designs can be utilized to decrease the chance that a correlation is causally confounded, and observational approaches can result in relatively reliable causal inferences [40]. Such approaches, like ours, emulate that of ideal randomized experiments as closely as possible [18] and attempt to cover many of theoretical criteria for causality. Criteria for causality were originally developed to show that tuberculosis was caused by a bacterium [14, 23]. Later, Hill [19] expanded on the idea of emulated randomized experiments by defining a set of criteria known as Bradford-Hill criteria. The idea behind these criteria is that pure correlation and pure causality are two ends of a spectrum, and the more criteria a relation can satisfy, the more likely it is capturing causality and vice-versa.

To understand the extent to which CLD coefficient captures causality is related to causality (i.e. how likely influence is the mechanism behind stickiness), we consider both: (1) it’s analogy to RCTs, and (2) the Bradford-Hill criteria it covers. Analogous to RCTs, CLD views the treatment as the language of friends in the past, and considers its effect on individual’s current language, where by the average language of the population represents the control group.^4^ In other words, on average, if people did not encounter their friends’ language, each person’s current language would be randomly drawn around the average current language of the population. If the language is sticky, encountering friends’ language in the past would change individual’s current language from the population’s towards the friends’. Note that we do not assume that encountering with friends’ language in the social network has to be the only medium of influence. The diffusion may happen through offline or other contexts, where one’s online friends are only a sample of the population of language they encounter.

Bradford Hill’s criteria for causation [19], includes 9 possible principles to establish a causal inference from an association relation, from which the CLD coefficient satisfies six. *Consistency or reproducibility*: we reproduce consistent CLD scores of word categories based on two separate networks. *Specificity*: we setup our experiments to rule out other possibilities of causation, e.g. considering intervals of 2 or 3 years removes the possibility of short term trends, or controlling for population’s language rules out the effect of overall language change on each specific word category. *Temporality*: the effect, *current* language (*L*^*c*^), happens after the cause, friends’ *past* language (*F*), in time. *Plausibility*: we show agreement with human judges. *Coherence*: there is no serious conflict with the relationship of people’s language and that of their surroundings. Finally, *Analogy*: We show the analogy of lexical diffusion with Tweets’ spread through predicting number of likes and retweets. Previous well-accepted studies have covered 5 to 7 criteria. For example ronksley2011association’s study of alcohol and cardiovascular disease outcomes covered 5 criteria, aghajafari2013association’s study of vitamin D level and pregnancy and neonatal outcomes covered 6 criteria, and hu2013resolved covered 7 criteria in their study of sugar-sweetened beverage consumption and the prevalence of obesity and obesity-related diseases.

Our work falls in line with this challenge of developing measures over observational data, where experiments are not possible, but which go beyond simple correlations providing evidence toward causality [2, 17, 36]. Never the less, because it is observational, we caution that a significant CLD coefficient, even along with careful study design, does not definitively prove causality.

## 5 EXPERIMENTS

### 5.1 Evaluation

We conduct several experiments to validate the utility of CLD-coefficients. We consider the predictive validity and reliability of CLD coefficients over: (1) two tweet-level tasks (predicting number of likes and number of retweets), (2) a user-level task (predicting friendship connections), and (3) a lexeme-level (association with human judgment of diffusion). We also conducted (4) an assessment of the stability of the coefficient as a lexical property by comparing its calculation over two independent training sets of users/tweets.

#### Likes & Retweets Prediction.

Retweeting and liking tweets are two key mechanisms of influence over the twitter network. While many factors come in to play for liking and retweeting, lexical diffusion should be associated with greater spread. We evaluate how well the number of likes and retweets of a tweet can be predicted by looking at only the CLD coefficient of the topics of the tweet. The CLD coefficient of the whole treat (*ψ*_*m*_) is taken as the aggregate of the CLD coefficient of the topics represented in the tweet as well as their prevalence:

(5) $$\psi_{m}=\underset{t}{\sum }p_{mt}*\psi t$$

where *m* is the message, *ψ*_*t*_ is the CLD coefficient of topic *t*, and *p*_*mt*_ represents the normalized frequency of topic *t* in message *m*. We end up with a single score per tweet, which we test for its lone-ability to predict retweets and likes by comparing it to: 1) number of likes, 2) number of retweets and 3) spread of tweet: a combination of number of likes and retweets of a tweet as shown in equation 6.

(6) $$S_{t}=Z\left(\text{log}\left(\left|r_{t}\right|+1\right)\right)+Z\left(\text{log}\left(\left|l_{t}\right|+1\right)\right)$$

where *S*_*t*_ is the intended spread of tweet for tweet *t*. ‖*r*_*t*_‖ and ‖*l*_*t*_‖ are the number of retweets and likes of tweet *t* correspondingly, and *Z*(.) yields the mean centered and standard deviation normalization of the scores (i.e. putting both scores on the same scale). The normalization (z-scoring) is per user, therefore the values of *S*_*t*_ are comparable among tweets of that specific user, and thus controls for the number of followers and other user-specific, non-lexical attributes that affect spread.

We used an “out-of-sample” collection of 31, 867 tweets, all of which were sent from 100 Twitter accounts not otherwise in our analyses. We ran a cross-validation regression that uses this CLD coefficient of tweet as predictor for each of those three labels separately. For comparison we consider two baselines. First is the sentiment of the tweet which has been shown to correlate with retweets [38]. Second is lexical emergence, for which we rank topics based on the degree they have risen in usage frequency from one snapshot to another. Then we calculate the message score similarly to equation 5. This follows work exploiting similar measures of lexical emergence to evaluate the spread of newly introduced words [15], something we expect our CLD coefficient, which is better suited for ordinary terms, to outperform.

Results shown in Table 1 demonstrate the CLD coefficient of the top 500 common topics, derived without any information about retweeting or likes, are in fact able to predict these metrics well beyond other metrics derived directly from the lexeme (absolute sentiment and lexical emergence scores) which have previously been shown predictive of likes and retweets. Results use a disattenuated Pearson correlation coefficient between the predicted and actual outcomes $r_{da}\left(x_{1},x_{2}\right)=\frac{r\left(x_{1},x_{2}\right)}{\sqrt{r\left(x_{1},x_{1}\right)r\left(x_{2},x_{2}\right)}}$ [8].^5^ Moving toward inclusion of less ordinary phenomena (with the advantage of higher recall), we repeated experiments using all the 2000 topics. As it is shown in Table 2 CLD coefficient still outperformed the baselines for all labels, but there is a decrease in pearson-*r* for CLD coefficient and lexical emergence. Such a decrease in performance supports the significance of being able to quantify diffusion of the most common (i.e. ordinary language).

#### Friendship Prediction.

People influence their friends’ behaviors in many different ways, including via language [1]. Examining the nodes (or people) of the network is another way to validate our measure of diffusion. Here, we predict the existence a friendship relation given sets of tweets for two nodes. We first randomly select 500 users from twitter, separate from our train users, forming test group. Then, corresponding to each user we randomly pick two other users: a friend and a stranger. The task is to see if we can predict which of these two potential friends is the actual friend. Putting the emphasis on CLD score, we make this selection by utilizing the *k* topics with highest CLD scores, then calculate weighted Jaccard similarity of the test user’s topic representation with each of his/her potential friends. The potential friend with higher Jaccard similarity, will be selected as the predicted friend of that test user.

**Figure 3:**
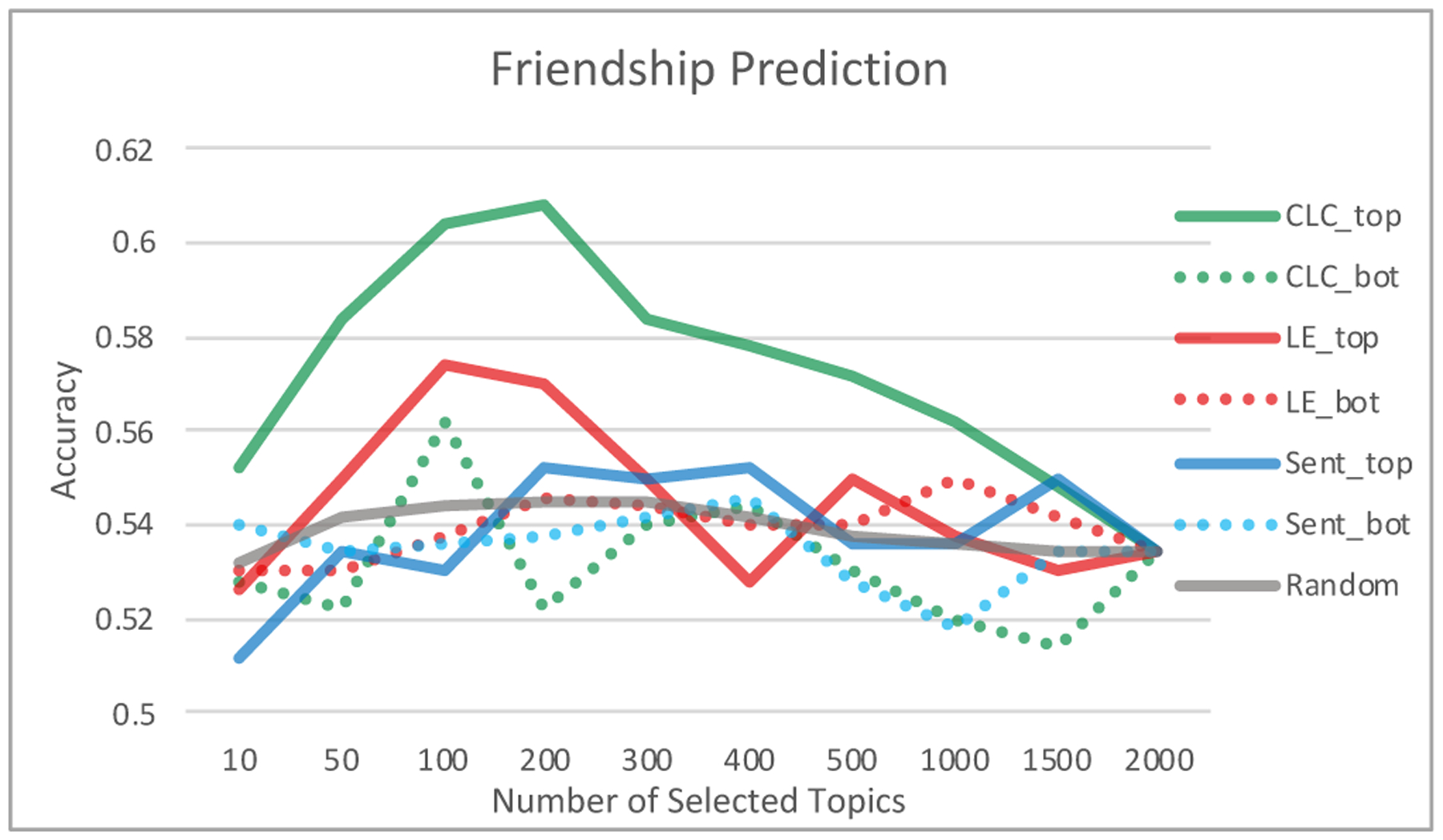
Friendship prediction using CLD coefficient vs. LE (Lexical Emergence), sentiment and random baselines. _*top* and _*bot*: only using topics with highest and lowest scores. ‘Random’ uses a random ordering for selecting top or bottom topics. While previous studies show friends language are similar, this suggests that such similarity is mostly among highly sticky topics.

Figure 3 depicts the accuracy of this method over different values of *k*, along with several different baselines (choosing top k from lexical emergence, sentiment, and randomly). We also considered selecting *k* topics with lowest scores for CLD coefficient which should be worse if our method is valid. This result shows an steady improvement in accuracy of CLD_top for *k* from 10 to 200 (approximately the top 10%), but then accuracy starts to fall off with minimum (chance) accuracy from use of all 2000 topics. We can see that CLD_top outperforms all the other 6 baselines. While past studies showed friends language are similar to each other [1], here we found that such similarity is facilitated mostly among topics with highest CLD coefficient.

#### Accuracy against Human Judgements.

The next experiment is a feature-level experiment that we conduct to compare the most and least diffusive topics according to human judgements. We asked a group of 5 graduate students with NLP-related expertise to compare 50 pairs of topics. We defined lexical diffusion for a topic as follows; *A topic (cluster of words) is considered to be diffusive in long-term*, *if people are more likely to use its words long time (a year or so) after they read tweets from their friends that contain the topic’s word(s*). For each pair of topics, annotators were shown the 10 most representative words from each, and asked to pick the topic that they assume to be more diffusive. Each pair was composed of a random selection of one topic from the top 50 and one from the bottom 50 according to CLD coefficient or, for comparison lexical emergence.

We next compare CLD coefficient with human judgements. Human judgements cannot be considered a gold-standard due to cognitive biases and difficulty of the task.^6^ Still, taken in the context of our previous objective evaluations, we consider human subjective judgements as a complimentary piece of evidence for whether our proposed metric is capturing diffusion. Specifically, the human task consisted of distinguishing the top and bottom 50 most diffusive topics. Here the inter-rater agreement, which is the average agreement of judges with each other is is 0.712 while the average agreement with majority vote is 0.828, This shows while the annotators are not compliant with each other, their individual agreement with majority is relatively high.

Table 3 demonstrates how CLD coefficient compares to the majority vote from the human judgements and accurately identifies what the majority found more diffusive in 92% of the cases, outperforming the average accuracy of annotators which is 83%. We see that CLD coefficient is well-aligned with the human notion of lexical diffusion while Lexical Emergence (based on frequency) is not able to capture lexical diffusion. Since diffusion or stickiness might be seen slightly different in raters’ minds, one might expect human judgement to be difficult to match based on the objectively defined CLD coefficient and ranking. However, by only comparing the top vs. bottom topics, in terms of CLD coefficient, we make the task coarser for humans to subjectively judge between sticky and non-sticky language. This provides a more reliable ground-truth for this experiment which is intended to compliment the more objective evaluations presented in the other experiments.

Qualitatively, our CLD coefficient is intended to capture its “stickiness” – to become used in greater amount by a person when they encounter it more often from a friend. We took a multi-modal approach to evaluate the validity and utility of our measure. We considered human judgments, utility in predicting likes and retweets, and utility in capturing friendships.

#### Reliability.

Evaluating the CLD coefficient on various prediction tasks, here we tend to find the reliability of our CLD coefficient. To do so, we run our method on the two disjoint networks that we explained in the Dataset section, to establish two sets of scores for the same group of 500 topics. We quantify reliability with two metrics, 1) Pearson-r correlation between these two sets of CLD coefficients 2) Jaccard similarity from the top 100 diffusive topics of each set of scores. Table 5 compares CLD coefficient to a random permutation of topics as the baseline. Both the correlation (above 0.7 is generally considered strong) and Jaccard similarity supports the idea that our measure is fairly consistent and that CLD coefficient can be seen as a property of language units, even though network structure is important on language diffusion.

### 5.2 Exploration

We conducted three exploratory experiments to demonstrate the use of CLD Coefficient for gaining insights about other phenomena: 1) Exploring CLD coefficient of LIWC’s word-categories, 2) Exploring topics with high CLD coefficient vs. low CLD coefficient and 3) Finding the relation of CLD coefficient with Big Five personality traits.

#### Word-category Lexica

We conducted an exploratory experiments to demonstrate the use of CLD Coefficient for gaining insights about lexical phenomena. Specifically, we looked at one of the most widely-used word category lexica, Linguistic Inquiry and Word Count (LIWC), which defines 74 categories of words with psychological relevance [33]. LIWC is widely used for work at the intersection of language and psychology (e.g. the 2001 version of the lexicon has over 4000 citations in scholar). We calculated the CLD coefficient of LIWC’s word-categories.^7^ Results in table 4 suggest: positive emotion is more sticky than negative; work is more likely to spread than any other domestic category like, leisure and home. Also the pronoun category has high CLD coefficient, with first-person plural pronouns (e.g. we) being more prone to spread than any other forms: singular first-persons (I) 2nd-persons (you), and third-person plurals (they). Lastly, categories of numbers and quantities do not seem to spread among friends. We also conducted a similar analysis over topics as well as an experiment on the relation of CLD coefficient with psychological language.

#### Sticky vs. non-sticky topics.

To get an idea what came out as scoring high or low in CLD coefficient, we depict the 5 topics with high CLD coefficient along with the 5 topics with low CLD coefficient in Figure 5. Sticky topics covered ‘meeting’, ‘government’, ‘job’, and ‘donation’. However, on the other side, non-sticky topics seemed to be more related to some curse words, negative thinking and emotion. Perhaps intuitively, global events, like ‘school orientation’ and ‘thanksgiving’ also came out with a low score.^8^ This corresponds with the idea that people either write about these events or not, not because their friends’ language but due to an external effect, the event itself.

#### Exploring Psychological Theory.

**Figure 4:**
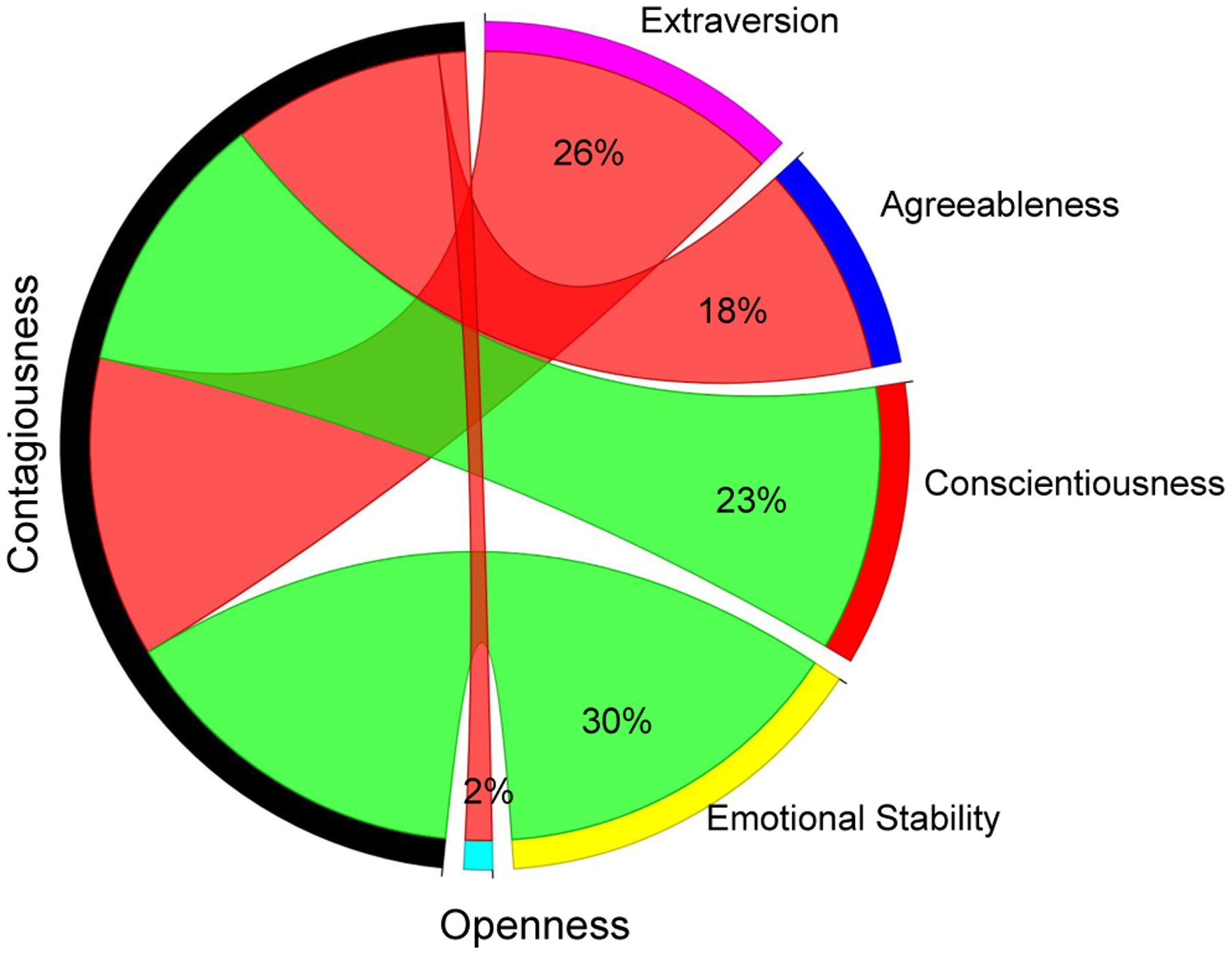
Big Five Personality traits vs. CLD coefficient: % variance explained from a multivariate OLS linear regression model over the 500 most common topics where the personality scores of topics from Park et al. (2015) are the predictors and CLD coefficient is the dependent variable. Red edge represents significant negative relationship, green edge represents significant positive relationship, and gray edge represents not significant relationship. In the context of all personality factors, emotionally stability scores were the most uniquely predictive of CLD coefficient while topics related to extraversion were less likely to be sticky in friendship communications.

**Figure 5:**
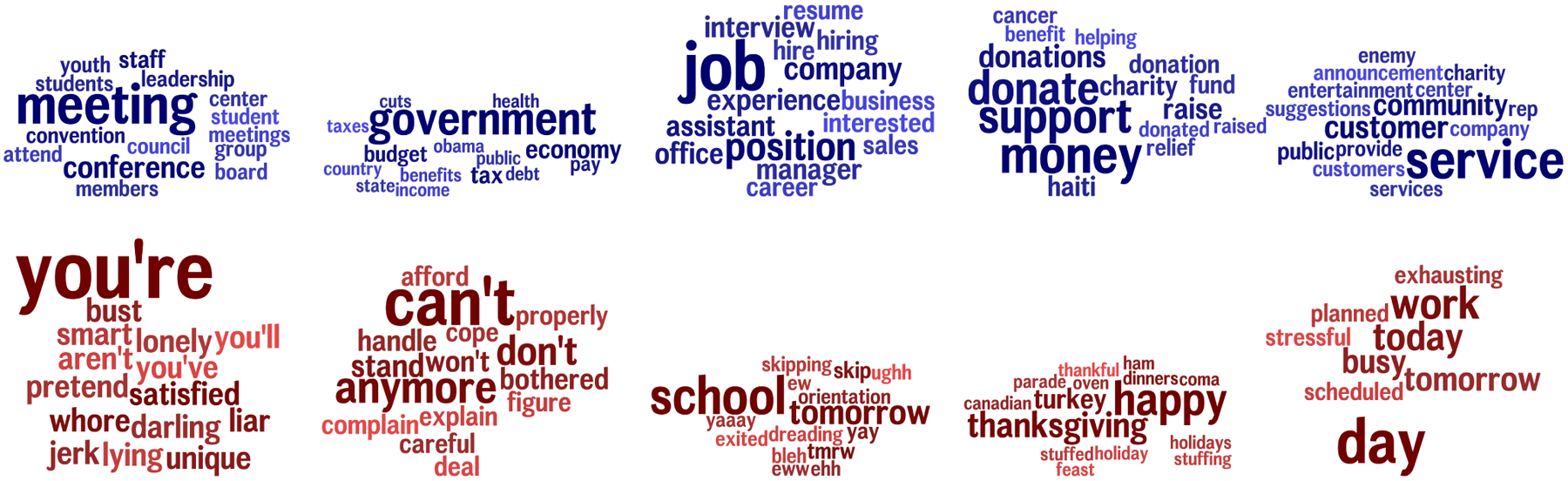
Five topics with high CLD coefficient (blue topics) vs. Five topics with low CLD coefficient (red topics): ‘meeting’, ‘government’, ‘job’, and ‘donation’ are among high CLD topics, while topics related to negative thinking and emotion, and global events, like ‘school orientation’ and ‘thanksgiving’ are among non-sticky ones.

Since CLD coefficient is intended to capture linguistic units that change under the influence of friends (people), we expect they should diverge along personality traits of people. we looked language associated with the Big Five personality traits [12], which consists of five factors: Openness to experience, Conscientiousness, Extraversion, Agreeableness, and Emotional Stability (inversely, also called neuroticism). Previous studies have released correlations between the social media topics and Big Five personality traits [37]. Here, we take the ranking of topics per factor and use a multiple regression to see how each personality factor, uniquely relates to CLD coefficient rankings. Results, depicted in figure 4, indeed revealed a variety of relationships, with some traits bearing virtually no relationship to CLD coefficient (openness) while others were strongly associated (emotional stability).

#### Adaptability of CLD..

We presented a novel measure to obtain CLD score, focused extensively on its evaluation. However, there are many avenues for extensions by injecting other parameters. For instance, one could incorporate link weights over a network by using the weighted average of friend’s language.^9^ A different way the approach is flexible is in the time resolution. While we focused on long-term language diffusion, CLD could be adapted to short-term diffusion by adjusting the time windows. This could aid in the study of short-term and long-term diffusion. As the introduction and evaluation of the approach, we focused on long-term frequency to make our validation scores reliable and well estimated.

## 6 CONCLUSION

We have presented and evaluated a novel method to capture frequency-based diffusion of lexical phenomena through friendship networks over time, while controlling for population effects. While work on dissemination of language over social networks has begun to provide a rich understanding of *lexical emergence*, it has left a gap in understanding of diffusion as a frequency-based process covering changes in *ordinary* lexical units (rather than introduction of *new* lexical units). We evaluated our CLD coefficient against indicators of influence (retweets, likes, and spread), as well as for distinguishing friends from non acquaintances, and versus human judgments. While past studies showed friends language are similar to each other, we have deduced that such similarity is facilitated mostly among the most sticky topics. Comparing CLD score of topics with their personality scores revealed topics associated with emotionally stability are more probable to be more likely to spread through friends, while those characteristic of agreeableness and extroversion have less chance to stick in their friends language use. Furthermore, CLD coefficient of lexical categories suggested new lexical diffusion information such as positive emotion being more sticky than negative or plural first-person pronouns (e.g. we) being more likely to spread than singular first-persons (e.g. I) and second-persons (e.g. You). We see this introduction and evaluation of the novel *CLD coefficient* as enabling greater computational study of diffusion of the ordinary language.

## Figures and Tables

**Table 1:** Disattenuated pearson-r for predicting number of likes and retweets, and spread of tweets (normalized sum of number of likes and retweets), using topic representation of tweets (with 500 most common topics).

	#Retweets	#Likes	Spread
Sentiment	0.250	0.119	0.233
Lexical Emergence	0.396	0.562	0.571
CLD coefficient	**0.643**	**0.693**	**0.771**

Comparing CLD coefficient of tweets vs. Sentiment (absolute value of sentiment score) and Lexical Emergence. Bold indicates significant improvement, through paired student t-test (*p* < 0.05), of CLD coefficient over both baselines

**Table 2:** Disattenuated pearson-r for predicting number of likes, number of retweets and spread of tweets, using all 2000 topics.

	#Retweets	#Likes	Spread
Sentiment	0.2	0.171	0.243
Lexical Emergence	0.4	0.428	0.464
CLD coefficient	**0.493**	**0.578**	**0.586**

Bold indicates significant improvement, through paired student t-test (*p* < 0.05), of CLD coefficient over both baselines.

**Table 3:** Accuracy of CLD coefficient and Lexical Emergence in matching human judgement of more diffusive topic: on average, CLD coefficient performed better than a single human.

Random Baseline	0.500	0.500
Average accuracy of humans	0.828	0.740
Accuracy of *CLD coefficient*	**0.920**	—
Accuracy of *LE*	—	0.540

Bold indicates significant improvement, through paired student t-test (*p* < 0.05). Lexical Emergence performed just shy above random baseline and much worse than average of human judges’ accuracy.

**Table 4:** Normalized CLD coefficient of LIWC lexica; Top 20 and bottom 10 categories are shown and color-coded from green (Positive values) to red (Negative).

Categoy	Score	Categoy	Score	Categoy	Score
Affiliation	1.00	Nets peak	0.56	Feel	−0.27
Drives	0.99	Pronoun	0.53	Quant	−0.29
We	0.97	Sad	0.45	Number	−0.30
Posemo	0.94	Bio	0.44	They	−0.38
Article	0.91	Ingest	0.43	Social	−0.43
Work	0.89	Negemo	0.41	Prep	−0.47
Percept	0.76	Adverb	0.40	Negate	−0.72
See	0.70	Verb	0.37	Future	−0.77
Space	0.67	Friend	0.34	Auxverb	−0.92
Power	0.64	Interrog	0.33	Present	−1.34

**Table 5:** Reliability test: comparing CLD coefficient of one network to another, and to random scores; Similarity of CLD coefficient of topics obtained from two separate networks shows CLD coefficient depends on the language units more than the network structure.

	Disjoint Network	Random
Pearson-*r*	0.85	0.00
Jaccard	0.64	0.12

## Footnotes

1 Each row of the matrix *L* is a vector representation of the language change of each individual.

2 The topics and their corresponding CLD coefficients are available here, https://github.com/mohammadzzamani/CLD_Score

3 for the purposes of this study we consider influence a case of causation

4 Note this assumes that a friend’s language has negligible effect on the population. However, should that assumption be wrong this will lead to an increase in false-negatives and less false-positives (i.e. miss potentially sticky lexeme rather than find fake lexeme), which we believe is the more reasonable compromise.

5 Disattentuated correlations account for measurement reliability – in this case the fact that the same tweet receives variations in the amount of likes and retweets not just based on the content but also based on the user. Here, because our CLD coefficient of the tweet is a linear combination (i.e. as an average) of CLD coefficients of the topics in the tweet, we disattenuate all results by the best linear prediction one could achieve (*r*(*x*_1_, *x*_1_) = *r*(*x*_2_, *x*_2_) = .15) using this data.

6 In other words, judgement of whether something is not a known task that distinguishes humans from machines

7 The CLD coefficient for LIWC lexica is available here, https://github.com/mohammadzzamani/CLD_Score

8 Our data for both *past* and *current* time spans includes several years, so they contain enough data for multiple iterations of yearly events

9 Our approach is also compatible with directed edges. In the case of Twitter, consider number of likes, retweets and mentions as the possible weights for directed friendship edges.
